# Immunomodulatory Effect of Polysaccharide from Fermented *Morinda citrifolia* L. (Noni) on RAW 264.7 Macrophage and Balb/c Mice

**DOI:** 10.3390/foods11131925

**Published:** 2022-06-28

**Authors:** Sun-Il Choi, Im-Joung La, Xionggao Han, Xiao Men, Se-Jeong Lee, Geon Oh, Hee-Yeon Kwon, Yong-Deok Kim, Geum-Su Seong, Seung-Hyung Kim, Ok-Hwan Lee

**Affiliations:** 1 Department of Food Biotechnology and Environmental Science, Kangwon National University, Chuncheon 24341, Korea; docgotack89@hanmail.net (S.-I.C.); xionggao414@hotmail.com (X.H.); menxiaodonglei@naver.com (X.M.); lsj9812@naver.com (S.-J.L.); dhrjs1@gmail.com (G.O.); 2 Atomy R&D Center, 3526, Charyeong-ro, Jeongan-myeon, Gongju-si 32511, Korea; imjna@atomyorot.kr; 3 NSTBIO Co., Ltd., 32, Songdogwahak-ro, Yeonsu-gu, Incheon 21984, Korea; khy@nstbio.co.kr (H.-Y.K.); ydkim@nstbio.co.kr (Y.-D.K.); 4 Korea Food Research Institute, 245 Nongsaengmyeong-ro, Iseo-myeon, Wanju-Gun 55365, Korea; gsseong824@nate.com; 5 Institute of Traditional Medicine & Bioscience, Daejeon University, Daejeon 34520, Korea; sksh518@dju.kr

**Keywords:** immunomodulation, polysaccharide, fermented *Morinda citrifolia* L., RAW 264.7, Balb/c mice

## Abstract

This study aims to determine the immunomodulatory effects of a polysaccharide fraction from fermented *M. citrifolia* L. (FMP) in RAW 264.7 macrophages and Balb/c mice. *M. citrifolia* was fermented for 72 h using *Lactobacillus brevis;* polysaccharides were extracted using ethanol precipitation. The RAW 264.7 cells exposed to FMP (50, 100, and 200 μg/mL) for 24 h showed increased NO production, proinflammatory cytokine (IL-1β, IL-6, and TNF-α) release, and COX-2 and iNOS protein expression. FMP (100, 200 mg/kg) and deacetylasperulosidic acid (DAA) (20 mg/kg) administered orally to Balb/c mice for 14 days upregulated NO production and NK cytotoxicity in abdominal cavity and spleen, respectively. Th1 and Th2 cytokines production and immune cell numbers increased in spleen, mesenteric lymph nodes (MLN), peritoneal exudate cells (PEC), Peyer’s patches (PP), and peripheral blood mononuclear cells (PBMC). Therefore, FMP containing DAA can be used as materials for health functional foods to enhance immune responses.

## 1. Introduction

With the acceleration of an aging society and spread of COVID-19, interest in health functional foods containing natural products that help improve immune function is increasing [1,2]. *Morinda citrifolia* L. (Rubiaceae) was discovered in Polynesia, India, and China around 2000 years ago. Polynesians used not only the flesh but also the peel, stem, leaf, and flower to heal wounds [3]. Organic acids, fatty acid esters, lignans, alkaloids, phenolic compounds, vitamins, and minerals have been reported as the bioactive compounds present in *M. citrifolia* [4], and are known to be effective against cardiovascular diseases, diabetes, cancer, and bacterial infections [5,6,7]. Therefore, researchers in the food and pharmaceutical industries are paying attention to the various effects of *M. citrifolia*, and its potential as a functional food ingredient has been investigated.

Food fermentation has the advantage of imparting a good taste, flavor, and texture to food through the action of microorganisms [8]. In addition, fermentation has been reported to improve the storage of food through lactic acid, acetic acid, and alcoholic fermentation [9]. It also improves the content of physiologically active substances, such as vitamins, essential fatty acids, and essential amino acids. It can even destroy toxic substances and improve digestion [10]. Fermentation is therefore widely used in the food industry. Despite its various functions, *M. citrifolia* has less value as a food ingredient because of its unique odor. However, Cheng et al. [11] and Wang et al. [12] reported that they could improve the smell of *M. citrifolia* fruit through fermentation. Furthermore, in our previous study, we confirmed that the content of deacetylasperulosidic acid (DAA), a marker compound of *M. citrifolia*, increased during fermentation [13]. The main phytochemical component of *M. citrifolia* is iridoid, of which DAA accounts for most [14]. DAA has been reported to inhibit lipoprotein oxidation [15] and DNA damage [16,17] through its antioxidant effect, and improve the atopic dermatitis and skin barrier function by modulating immune balance [18,19]. In addition, the protein expression of cyclooxygenase-2 (COX-2), IL-1β production, interleukin (IL)-6, and production of nitric oxide (NO) in RAW 264.7 cells treated with fermented *M. citrifolia* was increased compared to the group treated with non-fermented *M. citrifolia* [20].

The immune system defends the body from external antigens such as bacteria, fungi, bacteria, and viruses, and dysfunction of the immune system can lead to various diseases [21]. To protect the body from these external antigens, it is divided into innate immunity, which is responsible for immediate defense; and adaptive immunity, which creates and remembers specific antibodies or cells against external antigens [22]. The innate immunity confers primary physical defense through epithelial cells and mucosal layers in the skin, and phagocytosis of macrophages, dendritic cells, monocytes, neutrophils, and natural killer cells (NK cells) [23]. Macrophages and dendritic cells are immune response initiators with phagocytic action against foreign antigens. They secrete cytokines and chemokines to increase the activity of innate immune cells and deliver foreign antigens to induce adaptive immunity [24]. NK cells secrete granule type substances (perforin and granzyme B) to directly remove foreign antigens and secrete cytokines to induce adaptive immunity [25]. Adaptive immunity consists of activation of immune B and T cells against antigens presented by phagocytes, and it is amplified by the action of immune globulins, chemokines, and cytokines expressed in immune cells [26]. Therefore, adaptive and innate immune responses have been shown to be regulated by chemokines, cytokines, and immune cells [27].

Polysaccharides are complex molecules composed of long chains of monosaccharide units joined by glycosidic bonds. Polysaccharides exist in living organisms, such as mushrooms, yeast, fruits, seaweeds, and grains, and they are currently being studied as bioactive compounds in the immune system. Recently, the biological properties of polysaccharides, such as antioxidant [28,29], anti-inflammatory [30], and antiviral have been reported [31]. Non-toxic, biodegradable, and biocompatible properties have also been reported [32]. Polysaccharides are one of the major bioactive components of fermented *M. citrifolia* [33]. Therefore, in this study, the immunomodulatory effects of a polysaccharide fraction from fermented *M. citrifolia* (FMP) were determined using RAW 264.7 macrophage and Balb/c mice, and its potential as a functional food ingredient using natural plant resources was confirmed.

## 2. Materials and Methods

### 2.1. Chemicals and Reagents

Deacetylasperulosidic acid (CAS Registry No. 14259-55-3; ≥98%) was obtained from AOBIOUS (Gloucester, MA, USA). Trypsin-EDTA, Penicillin/streptomycin (P/S), Dulbecco’s modified Eagle’s medium (DMEM), phosphate-buffered saline (PBS, pH 7.4), and fetal bovine serum (FBS) were purchased from Gibco (Gaithersburg, MD, USA). Lipopolysaccharides (LPS), Sodium nitrite, and sulfanilamide were obtained from Sigma-Aldrich Co. (St. Louis, MO, USA), and N-(1-naphtyl) ethylene diamine dihydrochloride was purchased from Waco Co. (Tokyo, Japan). All antibodies and cytokine assay kits used for flow cytometry and immunoblotting analyses were obtained from Cell Signaling Technology (Danvers, MA, USA) and R&D Systems (St. Louis, MO, USA), respectively.

### 2.2. Sample Preparation

*M. citrifolia* harvested from the island of Java (Indonesia), was provided by NST Bio Co., Ltd. (Incheon, Korea). *M. citrifolia* was identified by Dr. Geum-Su Seong at the Korea Food Research Institute (Wanju, Korea). FMP was prepared by the following method: *M. citrifolia* (1000 kg) was fermented using 0.2% *Lactobacillus brevis* (NST707) for 2 weeks at 37 ± 2 °C. The ferment was filtered to eliminate fruit particles and debris and concentrated to obtain fermented *M. citrifolia* (10 Birx). The concentrated product (180 L) was mixed with 95% ethanol (720 L). After homogenization, a polysaccharide precipitate was obtained by incubation at 24 °C for 24 h. Purified water (100 L) was added to completely dissolve the polysaccharide (7–8 Brix) and freeze-dried to obtain FMP sample. The fermented red ginseng extract (Geumsan, Korea), used as a positive control, was identified and provided by Dr. Geum-Su Seong. Red ginseng (100 g) was fermented using 2% *Lactobacillus plantarum* for 24 h at 37 °C in 1000 mL of distilled water. The ferment was filtered and freeze-dried to obtain FRG. All samples were sonicated for 10 min in distilled water and filtered through a 0.22 μm PVDF filter (Millipore, Bedford, MA, USA).

### 2.3. Cell Culture and Cell Viability

The macrophage cell line RAW 264.7, was obtained from the American Type Culture Collection (ATCC, TIB-71, Manassas, VA, USA). The cells were cultured in high-glucose DMEM supplemented with 10% FBS. Equal numbers of cells (1 × 10^5^ cells/well) were incubated in a 5% CO_2_ atmosphere at 37 °C in a 96-well plate and treated with FMP and DAA for 24 h. XTT/PMS assay (WelGene, Seoul, Korea) was used to determine the cell viability according to the manufacturer’s protocol and was expressed as the percentage of absorbance of the untreated control at 450 nm.

### 2.4. Animal and Experimental Design

Eight-week-old male Balb/c mice were provided by Orient Bio (Seongnam, Korea) and maintained in a climate-controlled environment (humidity 55% ± 10% and temperature 23 ± 2 °C) with 12/12 h light/dark cycles and free access to food and water (2018S Rodent Diet, Minneapolis, IN, USA) for 1 week. Mice (n = 25) were divided into the following five groups (n = 5/group): group 1, Balb/c normal diet control (NC); group 2, normal diet + FRG (200 mg/kg); group 3, normal diet + FMP (100 mg/kg); group 4, normal diet + FMP (200 mg/kg); and group 5, normal diet + DAA (20 mg/kg). For oral administration, FRG, FMP, and DAA were dissolved in PBS and administered once a day for a total of two weeks. This study was approved by the Institutional Animal Care and Use Committee of Kangwon National University (approval number: KW-210802-1).

### 2.5. Measurements of Nitrite (NO) Production

RAW 264.7 cells were incubated with FMP (50, 100, and 200 μg/mL) and DAA (80 μM) for 24 h, and 100 μL of each sample culture medium was reacted with 100 μL of Griess reagent solution [1:1 mixture of 0.1% N-(1-naphtyl) ethylenediamine dihydrochloride in distilled water and 1% Sulfanilamide in 5% phosphoric acid (H_3_PO_4_)] for 10 min. The absorbance of the reactant was measured at 550 nm and a standard curve was used for calculations with sodium nitrite (NaNO_2_).

Two days before the last sample administration (day 12), peritoneal exudate cells (PEC) were elicited by injecting 4.5 mL of 2% starch saline into the peritoneal cavity of Balb/c mice for 48 h. After an abdomen massage for 30 s, PEC were harvested from the peritoneal fluid, lavage was performed (cold 1 × PBS), and then centrifugation at 400× *g* for 5 min at 4 °C. The PEC pellet was suspended in RPMI-1640 medium supplemented with 10% FBS and incubated in a 5% CO_2_ atmosphere at 37 °C for attachment of macrophages, for 2 h. Subsequently, the supernatant and suspended cells were removed and further incubated for 72 h. Next, 100 μL of each sample culture medium was reacted with 100 μL of Griess reagent solution for 10 min, and the absorbance of the reactant was measured at 550 nm.

### 2.6. Immunoblotting Analysis

RAW 264.7 cells were incubated with FMP and DAA for 24 h. Whole cells were lysed with a protein lysis buffer. Protein (30 μg) samples were separated by 10% SDS-PAGE and transferred to PVDF membranes. After blocking with 5% skimmed milk, the membranes were incubated with primary antibodies (1:1000) for 12 h at 4 °C and secondary antibodies (1:2000) for 1 h at 24 °C. Proteins were analyzed using ChemiDoc imaging system (Bio-Rad Laboratories, Hercules, CA, USA).

### 2.7. Measurements of Cytokines Production

RAW 264.7 cells were incubated with FMP and DAA for 24 h and centrifuged at 500× *g* for 5 min to obtain the culture medium. The levels of IL-6, IL-1β, and tumor necrosis factor alpha (TNF-α) in the supernatant were determined using an ELISA kit (R&D Systems) according to the manufacturer’s protocol.

The spleens isolated from the mice were washed with RPMI-1640 medium supplemented with 10% FBS, and a cell suspension was prepared by homogenizing and passing through a 70 μm cell strainer. Red blood cells (RBCs) were lysed from the spleen cell suspension with ACK lysing buffer. The cells were then washed with RPMI-1640 medium supplemented with 10% FBS. The obtained splenocytes were incubated in a 5% CO_2_ atmosphere at 37 °C for 24 h and then centrifuged at 500× *g* for 5 min to obtain the culture medium. Cytokines, including IL-2, IL-4, IL-10, IL-12, and interferon (IFN)-γ in the supernatant were determined using an ELISA kit (R&D Systems) according to the manufacturer’s protocol.

### 2.8. Measurements of NK Cell Cytotoxicity

Natural killer (NK) cell cytotoxicity was determined using a cytotox96 lactate dehydrogenase (LDH)-release assay kit (Promega, Madison, WI, USA) according to the manufacturer’s protocol. Using YAC-1 cells as the target cells, the ratio of the effector cells (obtained splenocytes) to target cells in each well was adjusted to 25:1 and 50:1 and cultured for 4 h. The supernatant (50 μL) was reacted with a chromogenic substrate to determine LDH activity, and the absorbance was measured at 490 nm using a microplate reader (Molecular Devices, Sunnyvale, CA, USA). NK cell cytotoxicity was calculated using the following formula: NK cell cytotoxicity (%) = (experimental effector spontaneous release-experimental target spontaneous release) / (target maximum release-target spontaneous release) × 100.

### 2.9. Isolation of Peripheral Blood Mononuclear Cells, Mesenteric Lymph Node Cells, Peritoneal Exudate Cells, and Peyer’s Patch Cells

To obtain peripheral blood mononuclear cells (PBMC), RBCs were removed from the blood samples using ACK lysing buffer and washed with RPMI-1640 medium supplemented with 10% FBS. To obtain total primary mesenteric lymph node (MLN) cells, MLN tissue was homogenized and filtered through a 70 μm cell strainer using the plunger of a 3-mL syringe. The cells were centrifuged at 400× *g* for 5 min at 4 °C and the pellet was resuspended in RPMI-1640 medium supplemented with 10% FBS. PEC were elicited by 2% starch saline for 48 h and harvested from the peritoneal fluid and lavage (cold 1 × PBS) containing PEC. The cells were centrifuged and resuspended in the media. To obtain Peyer’s patch (PP) cells, PPs were isolated from the surface of the small intestine, homogenized, and filtered through a 70 μm cell strainer. All isolated primary cells were used for the flow cytometric analysis.

### 2.10. Flow Cytometry Analysis

Isolated cells (PBMC, MLN, PEC, and PP) were stained with fluorescent-conjugated antibodies (anti-CD3, -CD4, -CD8, -CD11, -CD19, -CD23, -CD25, -CD49, -CD206, -F4/80, -Gr-1, and -SiglecF) in staining buffer (PBS with 3% FBS and 0.1% NaN_3_) for 10 min on ice. The stained cells were analyzed using FACS Calibur (BD Biosciences, San Jose, CA, USA) with Cell Quest software (version 6.0, BD Biosciences) and expressed as a relative percentage to the total cell number.

### 2.11. Statistical Analysis

Data are expressed as mean ± standard deviation (SD) for the in vitro study (RAW 264.7 cells), and standard error (SE) for the in vivo study (Balb/c mice). *p* values were calculated using one-way ANOVA with Dunnett’s post hoc comparisons using SPSS (version 24.0; IBM Corp., Armonk, NY, USA).

## 3. Results

### 3.1. Effect of FMP on RAW 264.7 Cell Viability

**Figure 1:**
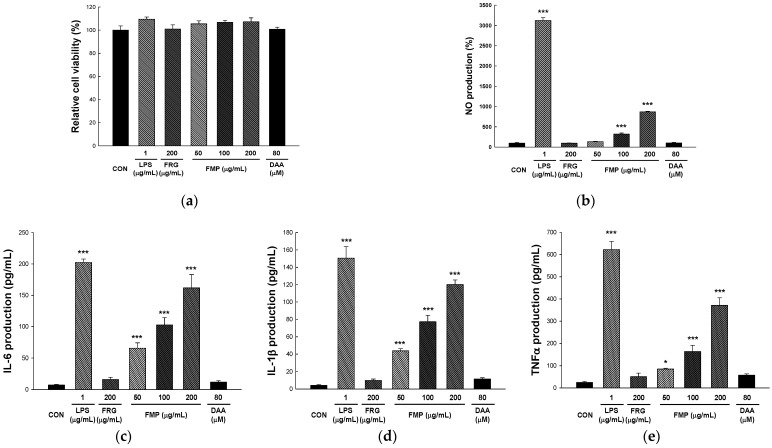
Effects of FMP on RAW 264.7 cell viability, NO production, and cytokine (IL-6, IL1β, and TNF-α) release in RAW 264.7 cells. The cells were incubated with FMP and DAA for 24 h. (**a**) Cell viability and (**b**) NO production were determined using an XTT assay and Griess reagent assay, respectively. (**c**) IL-6, (**d**) IL-1β, and (**e**) TNF-α production in RAW 264.7 cell culture supernatant was determined using an ELISA kit. All values are expressed as the mean ± standard deviation. * *p <* 0.05 and *** *p <* 0.001 vs. no treatment group. LPS, lipopolysaccharides; FRG, fermented red ginseng extract; FMP, fermented *M. citrifolia* polysaccharide; DAA, deacetylasperulosidic acid.

An XTT assay was performed to determine the effect of FMP on the viability of the RAW 264.7 cells. XTT assay uses the principle of generating dark red formazan by the cleavage of XTT by the mitochondrial dehydrogenase enzyme in living cells [34]. As shown in Figure 1a, FMP did not affect cell viability at 50, 100, and 200 µg/mL when compared with control (105.45 ± 2.64%, 106.78 ± 1.75%, and 107.30 ± 3.35%, respectively).

### 3.2. Effect of FMP on NO Production in RAW 264.7 Cells

The Griess reagent assay is a method for measuring the absorbance of an azo compound formed by reacting sulfanilamide (Griess reagent 1) and N-1-naphylethylenediamine (Griess reagent 2) with nitrite [35]. As shown in Figure 1b, NO production was not affected by treatment with DAA or FRG. However, the 50, 100, and 200 µg/mL FMP-treated groups showed significantly upregulated NO production in a dose-dependent manner compared to the untreated group (134.97 ± 6.03%, 321.82 ± 25.87%, and 869.95 ± 15.42%, respectively).

### 3.3. Effect of FMP on Cytokine Production in RAW 264.7 Cells

IL-6, IL-1β, and TNF-α are key cytokines that regulate immune response to infections, wounds, and various antigenic stimuli. They are produced not only by macrophages but also by various immune cells such as epithelial cells, monocytes, neutrophils, NK cells, T cells, and B cells [36]. To determine the effect of FMP on cytokine production, RAW 264.7 cells were incubated with FMP for 24 h and the levels of cytokines was measured in the culture supernatants using an ELISA kit. As shown in Figure 1c–e, FMP upregulated the expression of TNF-α, IL-1β, and IL-6 in a dose-dependent manner. In addition, it was confirmed that the FMP-treated group showed a significant increase in cytokine production compared to FRG, which demonstrated an immune-enhancing effect, and it is registered in Korea as a health functional food ingredient.

### 3.4. Effect of FMP on COX-2 and iNOS Protein Expression in RAW 264.7 Cells

**Figure 2:**
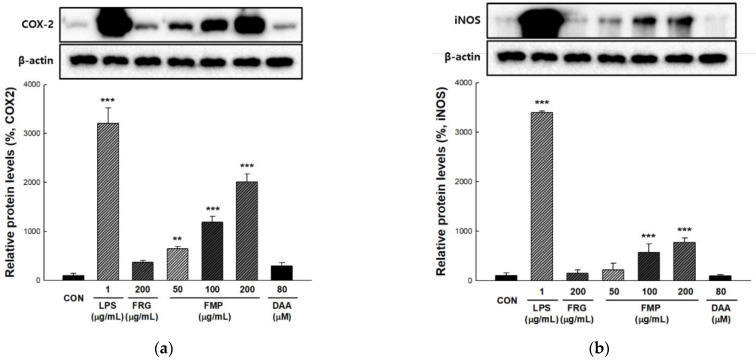
Effects of FMP on COX-2 and iNOS protein expression in RAW 264.7 cells. The cells were incubated with FMP and DAA for 24 h and harvested. The protein expression of (**a**) COX-2 and (**b**) iNOS was determined using immunoblotting. The relative protein expression levels were densitometrically quantified with β-actin. All values are expressed as the mean ± standard deviation. ** *p <* 0.01 and *** *p <* 0.001 vs. no treatment group. LPS, lipopolysaccharides; FRG, fermented red ginseng extract; FMP, fermented *M. citrifolia* polysaccharide; DAA, deacetylasperulosidic acid.

COX-2 converts arachidonic acid to prostaglandin E2 (PGE2) and modulates immune function by producing cytokines [37]. Inducible nitric oxide synthase (iNOS) contributes to pathogen death through NO produced by oxidizing l-arginine, while NO exhibits immunomodulatory roles, such as T cell activity regulation [38]. To determine the effect of FMP on the activation of COX-2 and iNOS, the total protein of FMP-treated RAW 264.7 cell was analyzed by immunoblotting. As shown in Figure 2a,b, COX-2 and iNOS protein expression was significantly increased by FMP treatment in a dose-dependent manner.

### 3.5. Effect of FMP on NO Production in Peritoneal Exudate Cells

**Figure 3:**
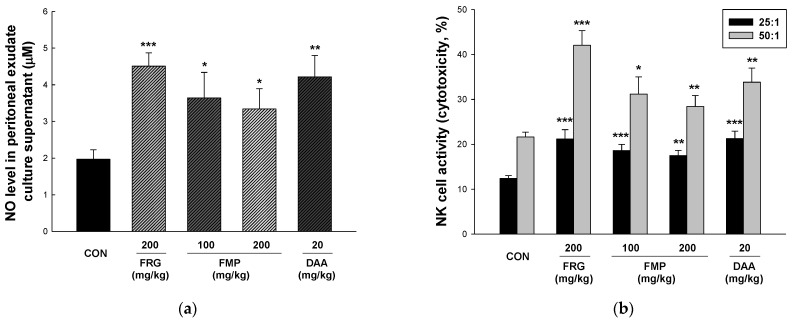
Effects of FMP on NO Production and NK cell cytotoxicity in peritoneal exudate cells and splenocytes from Balb/c mice, respectively. After oral administration for 12 days, peritoneal exudate cells were elicited by injecting 2% starch saline for 48 h and harvested. (**a**) NO production was determined using a Griess reagent assay. The splenocytes were co-cultured with target cells (YAC-1) for 4 h with a ratio of effector to target cells of 25:1 and 50:1. (**b**) NK cell cytotoxicity was determined using a LDH activity assay. All values are expressed as the mean ± standard error. * *p* < 0.05, ** *p* < 0.01, and *** *p* < 0.001 vs. no treatment group. FRG, fermented red ginseng extract; FMP, fermented *M. citrifolia* polysaccharide; DAA, deacetylasperulosidic acid.

The peritoneal cavity is surrounded by a membrane and filled with organs and fluids. Peritoneal fluid harbors a number of immune cells such as T and B cells, and macrophages [39]. Tissue macrophages play an important role in the immune response to tissue damage, pathogenic infections, and maintenance of tissue homeostasis [40]. To obtain peritoneal exudate macrophages, peritoneal exudate cells were elicited by intraperitoneal injection of 2% starch-saline. After 48 h, the suspended cells were removed from PEC to obtain macrophages, and NO production was measured using the Griess reagent assay. As shown in Figure 3a, the FMP-treated groups showed significantly upregulated NO production compared with the untreated group. In addition, NO production was significantly increased in Balb/c mice treated with DAA, in contrast to that in RAW 264.7 cells.

### 3.6. Effect of FMP on NK Cytotoxicity Activity

NK cells directly destroy infected cells or viruses without specific antigens and contribute to adaptive immunity by stimulating T cells induced by cytokine secretion [41]. For NK cytotoxic activity, effector cells (splenocytes) and target (YAC-1) cells were cultured at ratios of 25:1 and 50:1 (E/T ratio), and LDH activity was used to assess cytotoxicity against target cells. As shown in Figure 3b, an E/T ratio-dependent increase in NK activity was observed. The cytotoxicity of NK cells was significantly enhanced in the FMP- and DAA-treated groups compared to that in the untreated group.

### 3.7. Effect of FMP on Cytokine Production in Cultured Splenocytes

**Figure 4:**
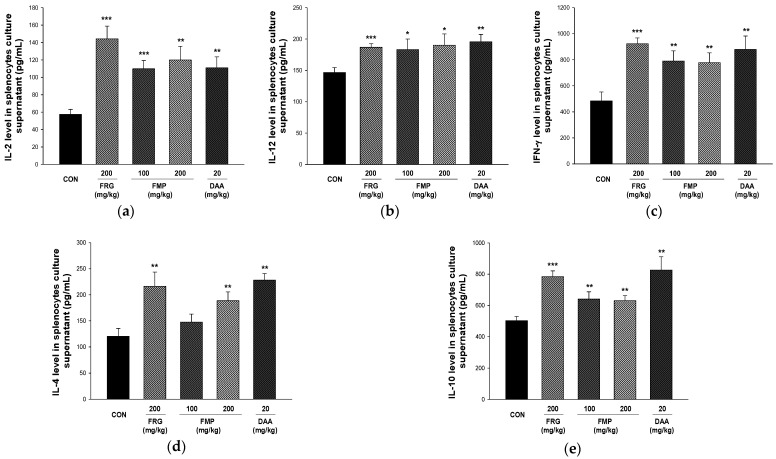
Effects of FMP on cytokine release in the medium containing splenocytes. The production of (**a**) IL-2, (**b**) IL-12, (**c**) IFN-γ, (**d**) IL-4, and (**e**) IL-10 were measured in the medium containing splenocytes, which was incubated for 48 h. All values are expressed as the mean ± standard error. * *p* < 0.05, ** *p* < 0.01, and *** *p* < 0.001 vs. no treatment group. FRG, fermented red ginseng extract; FMP, fermented *M. citrifolia* polysaccharide; DAA, deacetylasperulosidic acid.

T helper 1 (Th1)-type cytokines, including TNF-α, IFN-γ, IL-12, and IL-2, induce a macrophage-dependent immune response, and Th2-type cytokines, including IL-10, IL-6, IL-5, and IL-4 induce antibody responses through activation of B-cells [42]. As shown in Figure 4, the production of Th1-type (IL-2, IL-12, and IFN-γ) and Th2-type cytokines (IL-4 and IL-10) was higher in the FMP-treated group than in the untreated group.

### 3.8. Effects of FMP on Absolute Number of Immune Cell Subtypes in Spleen, Mesenteric Lymph Nodes, Peritoneal Exudate Cells and Peyer’s Patch Cells

Various types of lymphocytes play a major role in the immune response. We determined the populations of immune cell subtypes in the spleen, mesenteric lymph nodes, peritoneal exudate cells, and Peyer’s patch cells (Table 1). Oral administration of FRG, FMP, and DAA increased the total cell number in spleen, mesenteric lymph nodes, peritoneal exudate cells, and Peyer’s patch cells. It is interesting to note that while the absolute numbers of CD3^+^, CD3^+^/CD49b^+^, CD4^+^, CD4^+^/CD25^+^, CD49b^+^/NKG2D^+^, and CD23^+^/B220^+^ cells increased in the FMP-treated group, there was no significant difference in the absolute number of CD19^+^ and CD8^+^ cells in spleen compared to that in the untreated group. In addition, the absolute number of lymphocytes (CD4^+^, CD8^+^, and CD4^+^/CD25^+^ cells in MLN; CD3^+^, CD19^+^, CD206^+^/CD11b^+^, and CD11c^+^/F4/80^+^ cells in PEC; CD4^+^ and CD49^+^/NKG2D^+^ cells in PP) were significantly higher in the FMP-treated group than in the untreated group.

### 3.9. Effects of FMP on Populations of Peripheral Blood Mononuclear Cells

**Figure 5:**
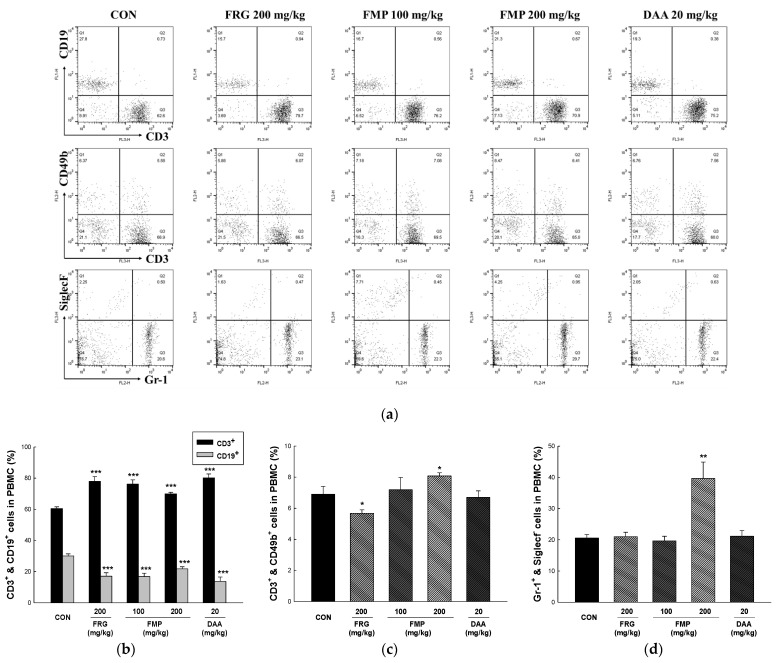
Effects of FMP on cell number in peripheral blood mononuclear cells of Balb/c mice. (**a**) Dot plot of representative fluorescence-activated cell sorting analysis of PBMCs and percentage of (**b**) CD3^+^ and CD19^+^, (**c**) CD3^+^ and CD49b^+^, (**d**) Gr-1 and SiglecF^-^ were determined using FACS Calibur two-color flow cytometer. All values are expressed as the mean ± standard error. * *p* < 0.05, ** *p* < 0.01 and *** *p* < 0.001 vs. no treatment group. FRG, fermented red ginseng extract; FMP, fermented *M. citrifolia* polysaccharide; DAA, deacetylasperulosidic acid.

PBMCs are blood cells of various immune cell types, including lymphocytes (B cells, T cells, and NK cells), monocytes, and dendritic cells [43]. As presented in Figure 5, it is interesting to note that while the population of CD3^+^ cells was significantly increased compared to that in untreated group, the population of CD19^+^ cells was significantly reduced in all treatment groups. In addition, the populations of CD3^+^/CD49b^+^ double-positive cells and Gr-1^+^/SiglecF^−^ increased in the FMP (200 mg/kg)-treated group. CD3^+^ cells are T cell co-receptor that is involved in activating both the cytotoxic T cell and CD19^+^ cells are B cells surface antigen. Therefore, FMP suggests that there is a possibility of regulating the immune balance in the state that asthma or allergic disease is induced due to the high activity of B cells in PBMC. However, further studies on the regulation of autoimmune disease induced by high levels of CD3+ cell numbers are needed.

## 4. Discussion

We aimed to investigate the feasibility of using a polysaccharide fraction from fermented *M. citrifolia* as an immunomodulatory functional material. Our previous study verified that fermented *M. citrifolia* significantly enhanced the immune response in RAW 264.7 macrophages [20], but a bioactive compound exhibiting immune activity could not be identified. Polysaccharides are macromolecules composed of monosaccharides and are important members of the biopolymer family. They are present in microorganisms, animals, and plants, and are involved in various physiological functions, such as neuroprotection, metabolic syndrome, and gastric protective, hypolipidemic, anti-obesity, antidiabetic, antimicrobial, antitumor, and immunoregulatory [44]. The immunomodulatory function of polysaccharides is mainly achieved by activating the adaptive and innate immune systems, specifically, lymphokine-activated killer cells, cytotoxic T cells, NK cells, B lymphocytes, T lymphocytes, and macrophages, by enhancing the production of immune-related antibodies and cytokines [45]. Ethanol precipitation is the most commonly used method for polysaccharide extraction. In addition, hydrolytic enzymes produced by microorganisms during fermentation break down the insoluble pectocellulosic cell wall into soluble polysaccharides to change the content and composition of the polysaccharides [46]. Botanical polysaccharides exhibit various physiological functions, but their biological activities differ depending on the composition and bonding structure of the monosaccharides [44]. The monosaccharide composition of the polysaccharide fraction of fermented *M. citrifolia* was 87.68 ± 3.30, 51.00 ± 0.38, and 69.92 ± 1.24 mg/g of glucose, galactose, and galacturonic acid, respectively (data not shown). However, further structural analyses and additional functional studies are required.

In general, immune organs are divided into central immune organs (thymus and bone marrow) and terminal immune organs (lymphoid tissues and spleen). Bone marrow is the site of growth, differentiation, and development of hematopoietic stem cells and produces various immune progenitor cells [47]. The thymus mainly induces T-cell differentiation and maturation and affects cell-mediated immunity [48]. The spleen is the largest immune organ in the body and can produce a large number of lymphocytes. It has a particularly important effect on the secretion of antibodies and production of B cells involved in humoral immunity [49]. Immune cells perform several important functions in the immune system. Immune cells such as dendritic cells, NK cells, monocytes, macrophages, T lymphocytes, and B lymphocytes are known to affect the adaptive and innate immune system through antigen recognition, antigen stimuli reception, and immune cell activation. Macrophages are regulatory immune cells directly remove pathogens and cancer cells through a non-specific immune response [50]. In addition, they are important antigen-presenting cells that secrete various cytokines, such as IL-1, TNF-α, IFN-γ, and NO, to activate the specific immune system [51]. Therefore, in this study, the immunomodulatory effects of FMP were evaluated using RAW 264.7 macrophages.

NO, produced by macrophages or neutrophils, has phagocytic activity, and inhibits pathogen proliferation by inhibiting the modification of pathogenic constituent molecules and the action of enzymes containing Fe-S [52]. Pathogens recognized by phagocytic receptors induce the production of various cytokines such as IL-12, IL-6, IL-1β, and TNF-α, and activate effector cells that present antigens to the adaptive and innate immune systems [53,54]. COX-2 is an enzyme that converts arachidonic acid to eicosanoids; it also plays an important role in innate immunity as a mediator of the adaptive immunity [55]. iNOS induces the production of NO and is used as an immune-related indicator, such as an immune signal transmitter and for autoimmune regulation [56]. In the present study, NO production and pro-inflammatory cytokine (TNF-α, IL-1β, and IL-6) release were increased by FMP treatment. COX-2 and iNOS protein expression levels were significantly increased by FMP treatment in a dose-dependent manner. Furthermore, FMP increased NK cell activity and NO production in Balb/c mice. These results suggested that FMP have the potential to enhance macrophage activity and innate immunity.

The adaptive immune response primarily clears exogenous antigens; it includes a humoral immune response which consists of antibodies produced by B and cytotoxic T cells, which have the ability to kill infected cells [57]. Immune responses are regulated by cytokines produced by antigen-presenting and Th cells. Th1 cells induce cellular immunity through Th1-type cytokine secretion, including IFN-γ, IL-12, and IL-2; while Th2 cells activate humoral immune responses through Th1-type cytokine secretion, including IL-13, IL-10, and IL-4 [58]. In the present study, FMP treatment increased the expression of both Th1-type (IL-2, IL-12, and IFN-γ) and Th2-type cytokines (IL-4 and IL-10). The immune response of Th2 cells increases the production of Th2-type cytokines, IgG1, and IgE. In contrast, Th1 and Th2 immune responses antagonize each other by producing IgG2 and Th1-type cytokines from Th1 cell immune responses [25,59]. In addition, an imbalance in which Th2 is dominant leads to immune hypersensitivity reactions (allergies, asthma, and atopy) [43]. In this study, it was confirmed that both Th1- and Th2-type cytokines were increased by FMP administration to Balb/c mice in a normal state. These results showed that the polysaccharides isolated from fermented *M. citrifolia* enhanced both cellular and humoral immune responses but did not affect the Th1 and Th2 balance. Furthermore, FMP effectively increased the number of total spleen, MLN, PEC, and PP cells, and significantly increased leukocytes (CD206^+^/CD11b^+^), monocytes (CD11c^+^/F4/80^+^), and lymphocytes, including T cells (CD3^+^, CD4^+^ and CD4^+^/CD25^+^), B cells (CD23^+^/B220^+^), and NK cells (CD3^+^/CD49b^+^ and CD49b^+^/NKG2D^+^) in spleen, MLN, PEC, and PP. In addition, administration of FMP increased the number of T cells (CD3^+^), NK cells (CD3^+^/CD49b^+^), and neutrophils (Gr-1^+^/siglecF^-^), while decreasing the number of B cells (CD19^+^) in PBMCs. Therefore, FMP upregulates immune responses by mediating both innate and adaptive immunity. However, in this study, the structure and molecular weight of the polysaccharides showing efficacy were not elucidated, and studies on the molecular mechanisms for enhancing the immunity of polysaccharides are required.

## 5. Conclusions

To summarize, this study demonstrated that FMP upregulated immune responses in RAW 264.7 macrophages and Balb/c mice. FMP increased NO production and proinflammatory cytokine release and activated COX-2 and iNOS in RAW 264.7 macrophages. Moreover, FMP upregulated NO production and NK cytotoxicity in the peritoneal cavity and spleen of Balb/c mice, respectively. Furthermore, FMP improved immune function by upregulating Th1 and Th2 cytokines and immune cells in the spleen, MLN, PEC, PP, and PBMC from Balb/c mice. These results indicate that FMP has immunomodulatory effects without affecting Th1 and Th2 immune balance in the normal state, not in the immunosuppressive and hypersensitive immune models. Therefore, bioactive compounds related to immune responses in fermented *M. citrifolia* were identified as low molecular weight phytochemicals as well as high molecular weight polysaccharides. Taken together, in the development of a functional food ingredient using fermented noni, FMP suggested the potential to be used as a marker and bioactive compound.

## Figures and Tables

**Table 1:** Effects of FMP on the spleen, mesenteric lymph node, peritoneal exudate, and Peyer’s patch immune absolute cell number in Balb/c mice.

Cell Phenotypes		CON	FRG 200 mg/kg	FMP 100 mg/kg	FMP 200 mg/kg	DAA 20 mg/kg
Spleen	Total spleen cells (×10^4^ cells)	345.33 ± 34.75	394.00 ± 22.69	440.00 ± 11.67 **	456.33 ± 57.59	456.33 ± 85.97
	CD3^+^ (×10^4^ cells)	174.82 ± 26.52	215.58 ± 19.08	266.53 ± 11.73 **	313.72 ± 56.22 *	267.24 ± 69.10
	CD19^+^ (×10^4^ cells)	142.87 ± 19.25	145.40 ± 13.64	137.25 ± 6.09	114.41 ± 21.20	140.59 ± 47.50
	CD3^+^ /CD49b^+^ (×10^4^ cells)	13.12 ± 1.04	14.92 ± 1.17	17.68 ± 1.78 *	13.74 ± 3.51	16.41 ± 3.16
	CD4^+^ (×104 cells)	102.16 ± 14.34	136.44 ± 13.67	149.06 ± 6.35 **	179.72 ± 32.57 *	147.05 ± 39.22
	CD8^+^ (×10^4^ cells)	65.62 ± 12.92	70.09 ± 9.07	83.14 ± 4.98	97.15 ± 13.13	85.91 ± 25.44
	CD4^+^/CD25^+^ (×10^4^ cells)	13.95 ± 1.71	20.51 ± 1.47 *	22.15 ± 0.87 ***	27.89 ± 6.87	22.87 ± 6.64
	CD49b^+^/NKG2D^+^ (×10^4^ cells)	11.54 ± 1.34	17.48 ± 2.19 *	16.35 ± 1.01 **	18.42 ± 2.89 *	16.89 ± 3.82
	CD23^+^/B220^+^ (×10^4^ cells)	28.83 ± 11.07	99.87 ± 11.08 ***	64.78 ± 5.08 **	88.25 ± 16.04 **	63.77 ± 15.91
MLN	Total mesenteric lymph node cells (×10^4^ cells)	259.33 ± 20.75	274.33 ± 23.71	435.33 ± 34.44 ***	401.00 ± 44.54 **	291.33 ± 22.63
	CD4^+^ (×10^4^ cells)	135.75 ± 15.14	161.18 ± 14.89	226.88 ± 24.11 **	221.95 ± 35.36 *	154.40 ± 19.31
	CD8^+^ (×10^4^ cells)	48.64 ± 4.13	48.79 ± 8.72	83.99 ± 10.02 **	81.43 ± 14.45 *	59.20 ± 8.07
	CD4^+^/CD25^+^ (×10^4^ cells)	14.09 ± 1.50	23.93 ± 5.20	27.27 ± 2.56 ***	26.19 ± 3.86 **	16.78 ± 2.17
PEC	Total peritoneal exudate cells (×10^4^ cells)	163.67 ± 18.97	238.33 ± 11.20 **	300.33 ± 23.56 ***	307.33 ± 37.59 **	244.00 ± 32.19 *
	CD3^+^ (×10^4^ cells)	19.06 ± 2.30	40.56 ± 6.50 **	29.65 ± 4.74 *	25.06 ± 2.39	28.06 ± 4.04
	CD19^+^ (×10^4^ cells)	92.18 ± 33.63	131.74 ± 14.53	197.16 ± 22.82 **	162.16 ± 26.51	145.54 ± 34.46
	CD3^+^/CD49^+^ (×10^4^ cells)	6.07 ± 3.86	6.39 ± 0.06	5.70 ± 1.24	10.95 ± 2.38	5.62 ± 0.85
	CD206^+^/CD11b^+^ (×10^4^ cells)	11.03 ± 1.29	13.66 ± 2.24	18.23 ± 1.29 **	22.10 ± 11.42	9.57 ± 0.96
	CD11c^+^/F4/80^+^ (×10^4^ cells)	5.73 ± 1.33	10.81 ± 1.01 **	13.04 ± 1.60 **	13.08 ± 1.15 ***	14.01 ± 3.98
PP	Total Peyer’s patch cells (×10^4^ cells)	267.33 ± 37.43	288.00 ± 10.26	342.67 ± 61.27	360.33 ± 21.52 *	478.67 ± 106.69
	CD4^+^ (×10^4^ cells)	54.91 ± 8.97	68.73 ± 1.24	69.53 ± 16.77	86.52 ± 7.05 **	122.70 ± 40.84
	CD8^+^ (×10^4^ cells)	12.61 ± 3.80	12.55 ± 0.36	14.73 ± 4.09	15.50 ± 3.99	26.66 ± 10.94
	CD49^+^/NKG2D^+^ (×10^4^ cells)	4.84 ± 1.24	3.69 ± 0.24	11.87 ± 3.63	8.86 ± 0.97 **	17.45 ± 6.53

* *p* < 0.05, ** *p* < 0.01, and *** *p* < 0.001 vs. control (CON) group. FRG, fermented red ginseng extract; FMP, fermented *M. citrifolia* polysaccharide; DAA, deacetylasperulosidic acid.

## Data Availability

The data that support the findings of this study are available on request from the corresponding author, Ok-Hwan Lee.
